# Raman Micro-Spectroscopy Can Be Used to Investigate the Developmental Stage of the Mouse Oocyte

**DOI:** 10.1371/journal.pone.0067972

**Published:** 2013-07-01

**Authors:** Bryony Davidson, Alison A. Murray, Alistair Elfick, Norah Spears

**Affiliations:** 1 School of Engineering, University of Edinburgh, Edinburgh, Scotland, United Kingdom; 2 Centre for Integrative Physiology, University of Edinburgh, Edinburgh, Scotland, United Kingdom; University Hospital of Münster, Germany

## Abstract

In recent years, the uptake of assisted reproductive techniques such as *in vitro* fertilisation has risen exponentially. However, there is much that is still not fully understood about the biochemical modifications that take place during the development and maturation of the oocyte. As such, it is essential to further the understanding of how oocyte manipulation during these procedures ultimately affects its developmental potential; yet, there are few methods currently available which are capable of providing a quantitative measure of oocyte quality. Raman spectroscopy enables investigation of the global biochemical profile of intact cells without the need for labelling. Here, Raman spectra were acquired from the ooplasm of mouse oocytes at various stages of development, from late pre-antral follicles, collected after *in vitro* maturation within their ovarian follicles and from unstimulated and stimulated ovulatory cycles. Using a combination of univariate and multivariate statistical methods, it was found that ooplasm lipid content could be used to discriminate between different stages of oocyte development. Furthermore, the spectral profiles of mature oocytes revealed that oocytes which have developed *in vitro* are protein-deficient when compared to *in vivo* grown oocytes. Finally, the ratio of two Raman peak intensities, namely 1605∶1447 cm^−1^, used as a proxy for the protein-to-lipid ratio of the ooplasm, was shown to be indicative of the oocyte’s quality. Together, results indicate that Raman spectroscopy may present an alternative analytical tool for investigating the biochemistry of oocyte developmental stage and quality.

## Introduction

The oocyte undergoes significant change during its development and maturation. Generally, these developmental processes are regarded as either nuclear or cytoplasmic maturation events, with both required for an oocyte to gain developmental competence, able to fully support fertilisation and embryo development. Nuclear maturation encompasses the successful completion of meiosis, whilst cytoplasmic maturation describes the extensive modification that the cytoplasm undergoes in order to support the resumption of meiosis and subsequently, successful fertilisation and embryo cleavage. During cytoplasmic maturation, the cytoplasm increases markedly in volume. More significant, though, is the accumulation of organelles and macromolecules and the cytoskeletal reorganisation, all of which contribute to the changing biochemical profile of the ooplasm during this period of maturation. In particular, marked increases in RNA expression, protein production, mitochondrial copy number and lipid deposits are all observed during oocyte cytoplasmic maturation [1]. Furthermore, oocyte-specific cortical granules appear and migrate to the periphery of the oocyte in preparation for recruitment in the polyspermy-block reaction. As such, it is evident that significant temporal biochemical variation exists within the oocyte during its development and maturation.

The oocyte gains the ability to complete post-ovulatory events while still enclosed in its follicle, doing so in a step-wise manner, so that it develops the capacity first to exit meiotic arrest and resume meiosis, then to undergo fertilisation and finally to fully support embryonic development [2]. Oocyte growth and development is not an independent process, instead a reciprocal relationship exists between the oocyte and surrounding granulosa cells, such that the oocyte regulates granulosa cell proliferation and function while, in turn, granulosa cells aid in the regulation of oocyte growth and development [3]. Ovulation of a species specific number of oocytes each oestrous or menstrual cycle is regulated by continual development of large numbers of follicles, which are then subjected to a series of selection processes such that only the required number will proceed to the pre-ovulatory stage [4]. As of yet, it is unknown if these selection processes occur solely to regulate the number of follicles developing to the pre-ovulatory stage, or if they also allow for the removal of intrinsically abnormal or poor quality follicles/oocytes. Yet, during ovarian stimulation cycles high levels of exogenous hormones are administered in order to support the continued development of surplus follicles, thereby selecting follicles that would otherwise have undergone atresia. Furthermore, high levels of circulating gonadotrophin have been reported to have a detrimental effect on follicles which have highly vascularised thecal layers in the later stages of maturation [5], [6].

New technologies are constantly being developed to overcome the obstacles faced by both female and male infertility. However, identification of oocyte maturity has been recognised as being one of the main obstacles to improving the efficiency of IVF treatments [7] and remains so. Consequently, the assessment of oocyte maturation and quality has attracted much attention, yet many of the techniques currently available lack the independent quantification required to significantly improve the efficiency of these treatments. Significant progress has been made in the assessment of nuclear maturation through the development of techniques such as birefringence imaging of the meiotic spindle, with this methodology reported to quantify developmental competence based upon the retardance of polarised light by the highly-structured spindle [8]–[10]. However, the conflicting results that have been reported using this technique [11] suggest the presence of factors other than meiotic maturity affecting subsequent developmental competence. In particular, cytoplasmic development has been identified as being pivotal to not only successful nuclear maturation, but also developmental competence. This work aimed to investigate if analysis of Raman spectroscopy can be used to determine the competency of oocytes, here using fixed oocytes to allow for extensive Raman analysis.

**Figure 1:**
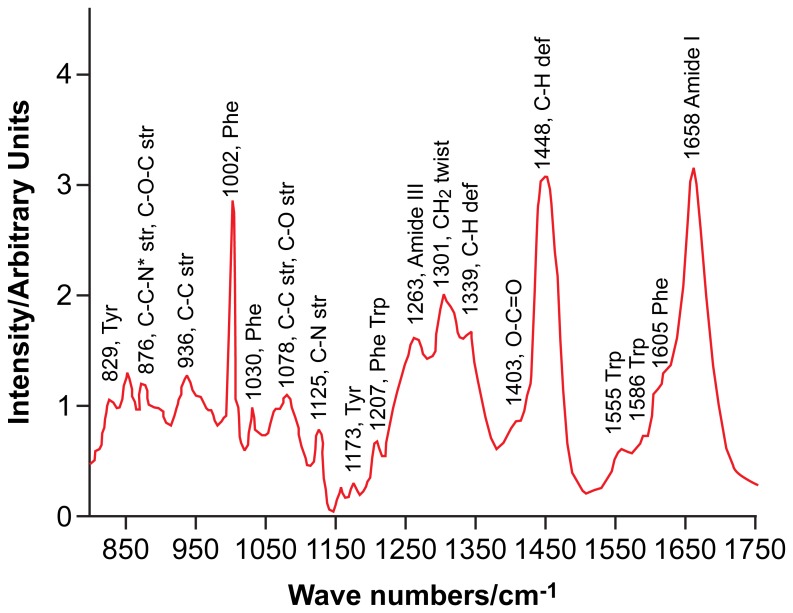
Analysis of Raman spectrum from a mature oocyte. Average Raman spectrum collected from the cytoplasm of a mature oocyte annotated to show the major peaks present in the wavenumber region 800–1760 cm^−1^, which can be attributed to specific proteins or lipids. Abbreviations str. and def. refer to vibrations arising from stretches and deformations, respectively.

Raman spectroscopy is a physicochemical fingerprinting technique in which laser photons interact with the bonds of a sample, stimulating their vibration, and in the process experiencing a shift in their energy levels (a schematic of the optical arrangement of the spectrometer and an illustration of peak assignments are presented in Figures S1 and S2). The energy shift of the photons can be measured and displayed as a spectrum; the intensity of photon count (arbitrary units) plotted against photon energy change expressed as wavenumbers (cm^−1^); Figure 1. As such, Raman spectroscopy provides a wealth of information regarding the presence of specific chemical moieties, which can then be interpreted to provide information for diagnostic algorithms, as evidenced by previous work in cancer identification [12]–[15], and, more relevantly, to analyse embryo culture medium in Assisted Reproductive techniques, to aid pregnancy outcome [16], [17]. This approach is particularly powerful when used in combination with multivariate statistical methods. Indeed, vibrational spectroscopy used in combination with principal component analysis (PCA) has been described for the discrimination of oocytes with intact germinal vesicles and those at MII collected after stimulated ovulation cycles [18], whilst Raman mapping informed by hierarchical cluster analysis has been used to image biochemical redistribution in maturing oocytes [19]. Raman offers the advantage of acquiring information regarding the biochemical constituents of a sample without labelling, on both fixed and unfixed samples. As such, this technique is capable of providing biochemical information which is complementary to that obtained using fluorescence microscopy.

In this work, Raman spectroscopy has been applied to the investigation of oocyte cytoplasm biochemistry. Specifically, the work compares oocytes from natural ovulations with those ovulated after superovulation regimes, and also examines how well follicle culture supports oocyte development. Overall, results report a direct quality assessment of oocytes using Raman spectroscopy.

## Materials and Methods

### Animals

Mice (F1 hybrid C57Bl/6J × CBA) were housed in an environmentally controlled room on a 14-hour light, 10-hour dark photoperiod, provided with food and water ad libitum. Superovulations were carried out by administering 5 i.u. pregnant mare serum gonadotrophin (PMSG, Intervet, UK), followed 47 hours later by 5 i.u. human chorionic gonadotrophin (hCG, Intervet, UK). Mice were culled by cervical dislocation. All animal work was in accordance with UK legal requirements, and the study was approved by the University of Edinburgh’s Local Ethical Review Committee.

### Sample Preparation

Four groups of oocytes were collected: immature (**IMM**); matured *in vitro* (**MIV**); matured *in vivo*, collected after natural ovulations (naturally ovulated: **NO**); and matured *in vivo* in response to a superovulatory regime (superovulated: **SO**).

#### Follicle dissection and culture

Late pre-antral ovarian follicles, 190±10 µm in diameter and which displayed good morphology, were dissected from the ovaries of 3-week old female mice. The resulting follicles were pooled and randomly divided into two groups: follicles in the first group were immediately ruptured to release immature oocytes (**IMM**; n = 39), whilst follicles in the second group were cultured to produce oocytes that have undergone a maturation process *in vitro* (**MIV**; n = 38). MIV oocytes were obtained from follicle cultured using a two-step process as follows. For the first culture process, intact follicles were cultured at 37°C and 5% CO_2_ for 6 days, in α-MEM supplemented with 5% v/v foetal bovine serum (FBS, Labtech, UK), 1 i.u. ml^−1^ recombinant human follicular stimulating hormone (r-FSH, gifted from Edinburgh IVF unit) and 180 µM ascorbic acid (SigmaAldrich, UK), as described in Murray et al. [6]. At the end of the six-day culture period, follicles had developed to the late antral stage, at which point they were ruptured and cumulus-oocyte-complexes (COCs) obtained. The second culture process cultured COCs for a further 24 hours at 37°C and 5% CO_2_ in α-MEM supplemented with 5% v/v FBS, 1 i.u. ml^−1^ r-FSH and 20 ng ml^−1^ epidermal growth factor (Boehringer). This two-step culture process has previously been shown to provide *in vitro* matured, fertilisable oocytes [6], [20].

#### Oocyte ovulation

Naturally ovulated (NO) oocytes were dissected from the fallopian tubes of mice, collected from unstimulated cycles in adult (6–12 week old) mice judged to be in oestrous by vaginal examination (**NO**; n = 38). Superovulated (SO) oocytes were collected from 6–12 week old mice previously injected with PMSG and hCG as detailed above, with oocytes dissected from the fallopian tubes of superovulated mice18 hours after hCG injection (**SO**; n = 59). SO oocytes were also graded from Grade 1 to Grade 5 for further analyses: oocyte grading was carried out after fixation (see section below), based on morphological appearance as outlined in Table 1.

**Table 1:** Description of morphological features used to grade oocytes.

Grade	Morphological Appearance
1	No cytoplasmic granularity and small perivitelline space
2	Presence of cytoplasm granularity
3	Large perivitelline space
4	Presence of cytoplasmic granularity and large perivitelline space
5	Fragmented oocyte

Oocytes were morphologically graded and given quality scores to facilitate investigation of the ability of the Raman fingerprint to quantify cell quality.

#### Preparation of oocytes for raman analysis

For all oocytes, cumulus cells were removed by pipetting in medium supplemented with 0.3 mg ml^−1^ hyaluronidase (SigmaAldrich, UK). Oocytes were washed thrice in phosphate buffered saline (PBS) before being fixed for 30 minutes in 4% w/v paraformaldehyde solution. Paraformaldehyde fixation was used, as paraformaldehyde-fixed and unfixed oocytes showed similar results following Raman analysis, compared to results following fixation with gluteraldehyde or ethanol, both of which caused significant modification of the spectra (see Figure S3 for comparison of various fixation protocols). After fixation, samples were washed thrice in PBS and stored at 4°C until analysis. For Raman analysis, oocytes were transferred to a 10 µl drop of PBS in a sample holder which consisted of a quartz coverslip (UQG Optics, UK) with cloning cylinder attached: the oocyte and PBS were then overlaid with 75 µl of silicone fluid (Dow Corning, UK).

### Raman Spectroscopy Measurements

All Raman measurements were collected using an InVia Raman spectrometer (Renishaw plc, UK) coupled to a DM IRB inverted microscope (Leica Microsystems, Germany). Illumination was delivered using a 785 nm laser (Toptica, Germany) and focused via a 40×/0.65 NA microscope objective (Leica Microsystems, Germany). For each oocyte, 101 individual spectra were collected at a constant spacing of 6 µm, all taken from within the zona pellucida, through the central focal plane of the oocyte. Each spectrum was integrated for 37 s over the spectral range 450–1790 cm^−1^ using a laser power at the sample of 35 mW. A spectrum of PBS buffer which surrounded the oocyte was also acquired using the same experimental conditions. Spectra of a polystyrene standard (SigmaAldrich, UK) and a neon light source (Renishaw plc, UK) were also taken to calibrate the wavenumber scale.

### Data Preprocessing and Extraction

All data preprocessing was performed using MATLAB and in-house written scripts. Firstly, data underwent cosmic ray removal and the wavelength scale was corrected using the neon and polystyrene spectra. The 101 spectra which were collected in a grid pattern across each sample were treated as a group and subjected to a preprocessing routine. Sample spectra and PBS spectrum were smoothed using a 3-point and 11-point boxcar function, respectively. The spectral contribution of the PBS was then subtracted using the first derivative orthogonal vector method suggested by Maquelin et al. [21], following which the spectra were returned to their integrated form. Autofluorescence background was subtracted by fitting a fourth order polynomial. Next, an average spectrum was generated by taking the arithmetic mean of the 101 processed spectra. Average spectra were truncated to the region 800–1760 cm^−1^ for the data analysis. Following this, each spectrum was offset-corrected, such that the minimum intensity was set to zero. Finally, each spectrum was normalised to its mean spectral intensity across all wavenumbers in order to account for fluctuations in laser intensity.

### Data Analysis and Statistics

All data and statistical analysis was performed using MATLAB 8.0 and the Statistics Toolbox (Mathworks, USA). Statistical tools employed are summarised below; greater detail may be found in the Supporting Information (see Text S1). In addition to univariate testing, multivariate statistical methods were adopted for data interpretation. High dimensionality is one of the major obstacles to statistical analysis of spectroscopic data. To simplify the task of analysing excessively large numbers of samples, data were subjected to dimension reduction by means of PCA. During PCA, each spectrum was assigned a score for each of a set of newly generated principal components (PCs), each of which described the contribution that the PC made to the original spectrum. Although PCA can readily illustrate variation between samples, its use in the classification of additional samples is limited by its methodology, whilst the application of a logistic regression algorithm, using intensity ratios directly extracted from each sample’s Raman spectrum, enabled the straightforward classification of additional samples. Canonical variate analysis (CVA), unlike PCA, is specifically designed to investigate the discrimination of samples based upon classification. Receiver operating characteristic (ROC) analysis allows the relationship between the sensitivity and specificity of a predictive model to be explored, such that an optimal cut-off point between these two parameters may be established.

#### Analysis of oocyte development *in vitro*

A preliminary investigation was carried out to determine if oocytes from cultured ovarian follicles (MIV) could be discriminated from the immature oocytes collected at the start of the culture period (IMM), with PCA performed on the data set consisting of all mean spectra derived from the IMM and MIV oocytes. Spectra from IMM and MIV oocytes were randomly allocated to either a training set or a validation set, with 60% of each group’s oocytes used for the training sets and 40% for the validation sets. Spectra from the training groups were then used to develop a logistic regression algorithm to classify oocytes as immature ( = 0) or mature ( = 1). Using a leave-one-out cross validation methodology combined with sequential forward feature selection, the model coefficients were optimised such that the deviance, as defined by −2 log likelihood ratio, of the model fit was minimised. The predictive ability of the algorithm was then tested using the validation data sets.

#### Comparison of oocyte development *in vivo* and *in vitro*

Naturally ovulated (NO) oocytes were compared with those developed *in vitro* (MIV) and with oocytes collected after a superovulation regime (SO). PCA was performed on the mean spectra derived from the three groups of oocytes. Subsequently, so as to further investigate the discrimination of the three groups, CVA was performed using the scores derived for the first seven principal components (PCs) and leave-one-out cross-validation, so that each sample was predicted once.

#### Analysis of quality assessment

Student’s t-test was used to identify intensity ratios of Raman peaks which were found to differ significantly (p = 0.01) between oocytes of Grade 1 and those of Grades 2–5. ROC analysis was used to define a discrimination threshold for oocyte quality based on a protein-to-lipid ratio defined from spectral data across the various groups.

## Results

### Biochemical Interpretation of the Oocyte’s Raman Spectrum

As illustrated by Figure 1, the Raman spectrum of the mouse oocyte is complex and displays a significant number of overlapping bands in the fingerprint region. Contributions mainly result from protein and lipid molecular vibrations. Strong peaks are seen in the region of the amide I and amide III vibrations at 1658 cm^−1^ and 1255 cm^−1^ respectively. The phenomenon of Raman band broadening is often the result of overlapping bands. This is particularly true of the amide I region, within which the location of this band is particularly sensitive to the secondary structure of the proteins investigated: the precise location can occur anywhere in the region 1645–1675 cm^−1^, with lower, intermediate and upper regions indicative of a α-helical, random coil and β-sheet secondary structures respectively. Furthermore, an overlap exists between the amide I band and the C = C stretch in unsaturated lipids and carbohydrates. Non-overlapping contributions resulting from amino acids with aromatic side chains are seen at 829, 856, 1002, 1030, 1173, 1207, 1605 and 1621 cm^−1^. The tyrosine doublet, which is observed at 829 and 856 cm^−1^, is the result of in-plane ring breathing, while the sharp characteristic band at 1002 cm^−1^, which is seen in the spectra of most biological samples, arises due to the symmetric ring breathing of phenylalanine. Peaks at 1030 and 1207 cm^−1^ occur due to the C-H in-plane stretch of phenylalanine and the aromatic-carbon stretch of both phenylalanine and tryptophan, respectively. Similarly, peaks which form the shoulder of the amide I band at 1605 and 1621 cm^−1^ are attributed to phenylalanine and tyrosine respectively. Furthermore, the non-overlapping contribution resulting from the CH_2_ twist, which occurs in lipid molecules, is seen at 1301 cm^−1^. Additionally, due to the complexity of the biochemical composition of the ooplasm, it is expected that overlapping bands will exist in its spectrum. Such bands occur in the region of 1420–1480 cm^−1^, corresponding to the C-H deformation in proteins, lipids and carbohydrates. Significant overlap also exists in the region below 1000 cm^−1^, where contributions from proteins and carbohydrates in particular are observed. Furthermore, in some spectra, bands arising from the molecular vibrations of nucleic acids are observed. In particular, contributions from the nucleic acid ring breathing and CH_2_ scissoring in the backbone are observed in the region 1350–1380 and 1410–1425 cm^−1^ respectively.

### Biochemical Differences between Oocytes at the Start and End of Follicle Culture

**Figure 2:**
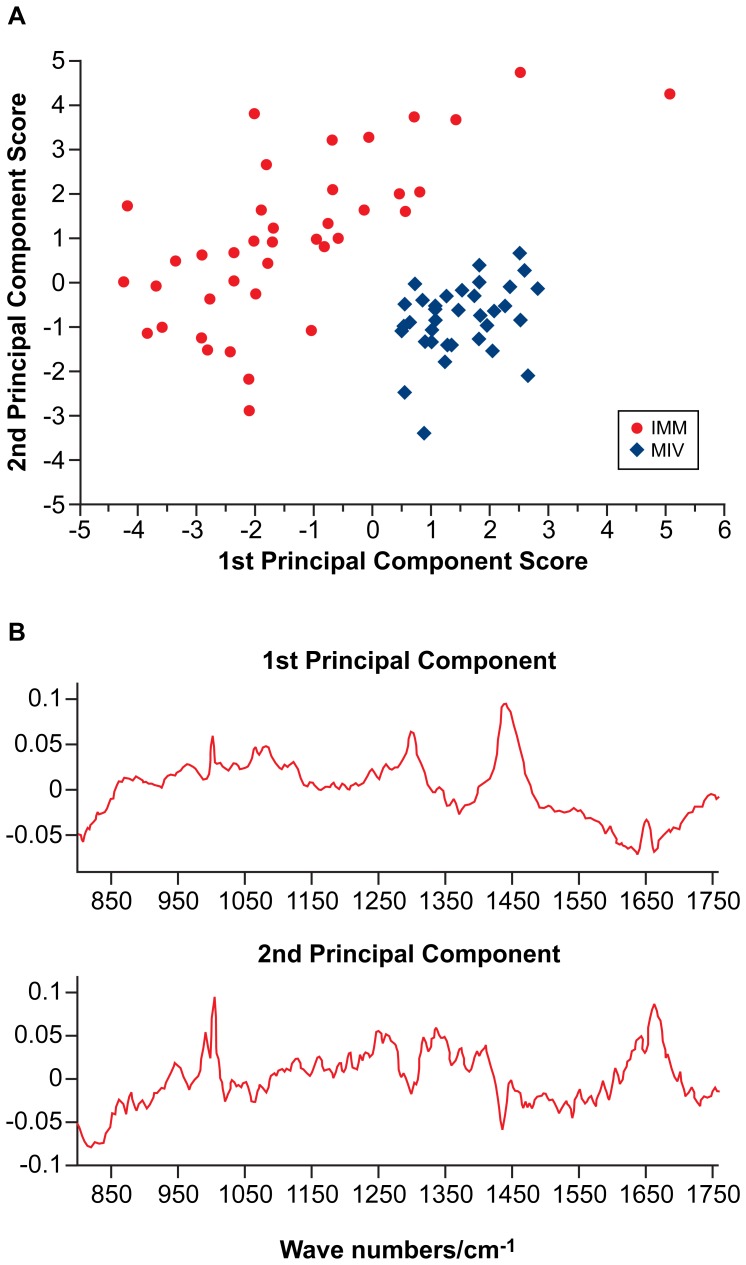
Principal component analysis of data from immature (IMM) oocytes and oocytes matured in vitro (MIV). (**A**): Biplot showing the scores of each datum obtained from IMM and MIV oocytes plotted against the first and second component axes generated during PCA. Separation of immature and mature oocytes is observed along both the first and second axis. (**B**): Loadings for the first two Principal Components generated for the immature and mature datasets.

Raman fingerprinting was successfully used to discriminate between oocytes at the start (immature: IMM) and end (matured *in vitro*: MIV) of the follicle culture period. PCA was performed on a data matrix that contained the 77 average Raman spectra collected from the two sample groups. Figure 2A depicts the scores of the first and second PC for each oocyte sampled. A clear distinction can be seen between IMM and MIV oocytes, with separation of the two groups along both the first and second PC. Although a spread of scores along the other significant PCs is observed, no distinction exists between the groups in these dimensions; instead they appear to describe inter-oocyte biochemical variance. Furthermore, an insight into the synchronicity of oocyte and follicular development can be gained from examining inter-oocyte variation within the two treatment groups; despite the fact that all IMM oocytes come from follicles within a narrow size-window (180–200 µm in diameter) compared to the range of follicle diameters at the end of culture (from around 300 to 450 µm in diameter: [6], [20]), a greater inter-oocyte variance is observed in PC scores within the IMM group compared to the tightly clustered MIV oocyte group scores (Fig. 2A). This suggests an asynchronicity between oocyte and follicular development at earlier stages of follicle development.

The nature of the biochemical variation between oocytes from the start and end of the follicle culture period can be revealed by examination of the PC loadings (Figure 2B): The first PC reveals positive loadings in the region 1050–1140, 1290–1310 and 1430–1465 cm^−1^, regions predominately associated with lipid bond vibrations, whilst negative loadings are observed in the region of protein aromatic residue vibrations and the amide I envelope at 1550–1700 cm^−1^. Conversely, the second PC, which also describes separation of the two sample groups, shows positive loadings associated with protein bond vibrations: specifically, positive contributions are observed in the region of the C-C stretch at 990 cm^−1^, the amide III band at 1240–1280 cm^−1^, the C-H deformation at 1340 cm^−1^ and the amide I envelope at 1600–1670 cm^−1^.

### Use of Raman Spectroscopy to Predict Oocyte Developmental Stage

**Figure 3:**
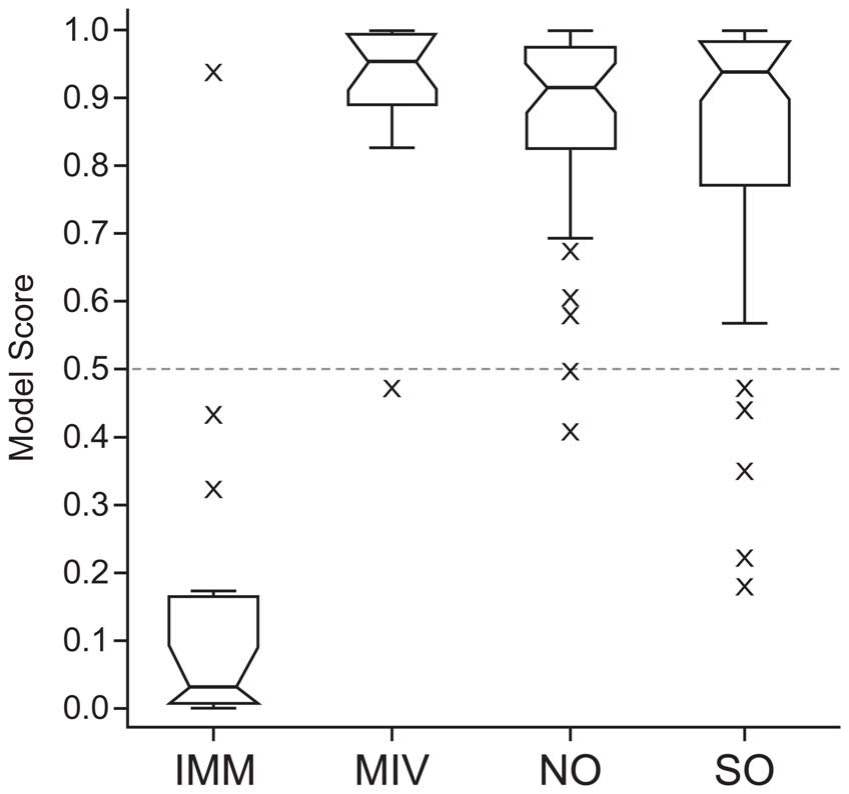
Box-plot showing predictive ability of logistic regression algorithm. Validation group consisted of IMM (immature: n = 15), MIV (matured in vitro: n = 15), NO (naturally ovulated: n = 38) and SO (superovulated: n = 59) oocytes. Within each box-plot, the narrow line shows the median, while upper and lower lines indicate first and third quartiles, with black whiskers extending 1.5 times the inter-quartile range in both directions: data outwith of this range are considered outliers, annotated by crosses.

The utility of oocyte assessment based on Raman spectroscopy is predicated on the ability to classify an unknown oocyte’s developmental stage with high sensitivity and specificity. A logistic regression algorithm was optimised using a training dataset (60% of the original data from the IMM and MIV groups). Blinded analysis of the model’s predictive ability was then performed using the independent validation set, which consisted of the remaining 40% of the data from the IMM and MIV groups. The model was capable of predicting whether oocytes were at an immature developmental stage (as would be expected of oocyte from late preantral follicles, the source of the IMM oocyte group), or had developed further (as would be expected of oocytes collected from follicles that had been grown *in vitro*, the source of the MIV oocyte group): using an unbiased classification cut-off of 0.5, data show that only 1 of 15 IMM oocytes was classified as mature, whilst only 1 of 15 MIV oocytes was classified as immature, equating to a sensitivity of 93.3% and specificity of 93.3% (Figure 3).

The classification was then tested further using oocytes collected after unstimulated (NO) and stimulated (SO) ovulation cycles. Significantly, although the model was constructed using oocytes which had developed *in vitro*, it was successfully used to classify *in vivo*, ovulated oocytes: only 2 of 38 NO oocytes and 6 of 59 SO oocytes were classified as immature (Figure 3); inclusion of these data results in only a minor reduction in overall sensitivity of the model to 92.0%.

### Analyses of Effect of Environmental Conditions on Oocyte Development

**Figure 4:**
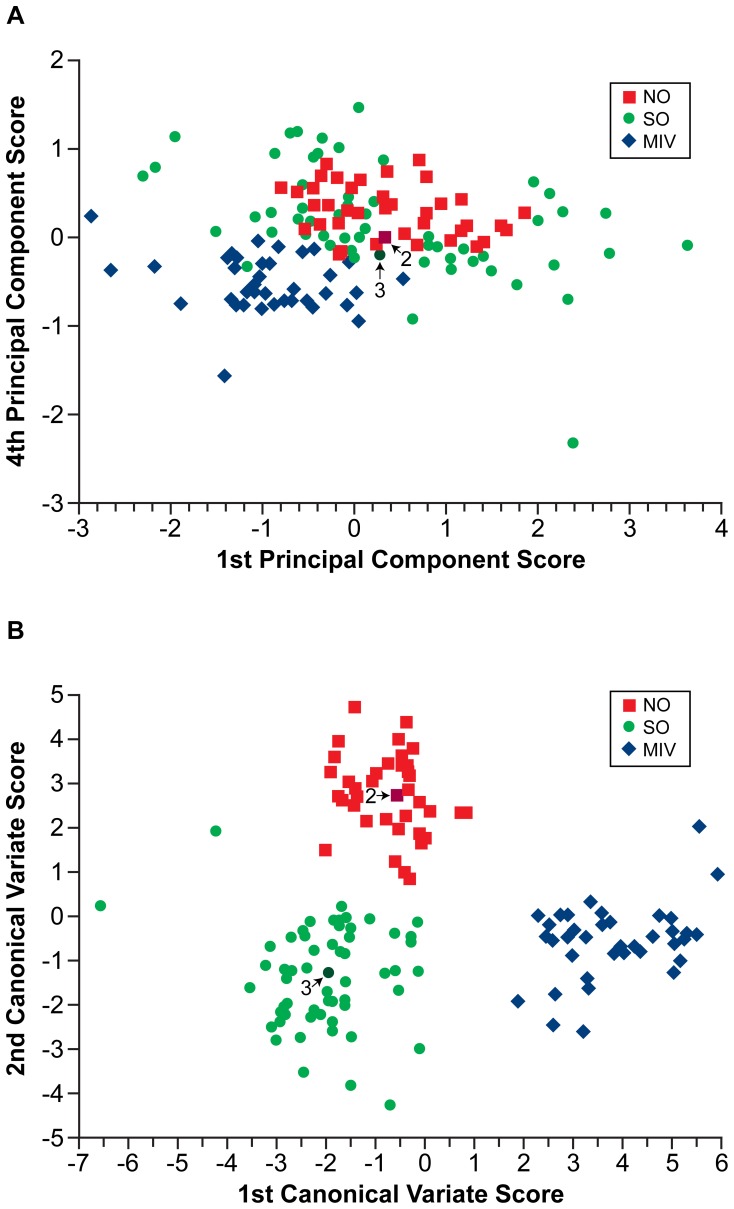
Principal component analysis and canonical variate analysis of data from oocytes. Scatter plot of oocytes that were naturally ovulated (NO), superovulated (SO) or matured in vitro (MIV), showing the scores of each datum against (**A**): the first and fourth component axes generated during PCA and (**B**): the first and second canonical variate axes generated during CVA.

Analysis of oocytes revealed differences depending on whether oocytes developed during a natural ovulatory cycle (NO), or during two very different manipulations, that of superovulation (SO), or after development in culture (MIV). PCA was performed on a data matrix containing the 135 average Raman spectra which had been collected from the three treatment groups (n**_NO_** = 38, n**_SO_** = 59, n**_MIV_** = 38). Figure 4A depicts the scores of the first and fourth PC for each oocyte sampled from the NO, SO and MIV groups. The scatter plot shows that a marked separation exists between the NO and SO groups and that of the MIV group along the fourth PC. Furthermore, it reveals a distinction between the NO and MIV groups along the first PC. Although a spread of scores along the other significant PCs is observed, no distinction between the groups exists in these dimensions. The group means for the NO and SO groups are very close to one another, whilst the MIV group mean appears distinct, although the scores of the SO group do overlap with both the NO and MIV groups. However, the variance of the scores for the SO group is greater than that of the NO or MIV groups, as demonstrated by the tight clustering of the latter groups. No within group clusters were observed for the different experimental replicates.

CVA was then performed using the scores from the first seven PCs, to limit the error contribution from within group variation; combined they described over 80% of the variance which existed in the original data. The results of the CVA revealed separation of both groups of oocytes ovulated after development *in vivo* (NO and SO) from the oocytes obtained at the end of follicle culture (MIV group), observed along the axis of the first canonical variate (CV), whilst separation of NO oocytes from the SO and MIV oocytes is observed along the axis of the second CV (Figure 4B). Results from the CVA confirmed those obtained from the initial PCA analysis, showing that the MIV group mean is distinct from both groups of oocytes that developed *in vivo* (the NO and SO groups). However, the CVA also revealed that oocytes collected after superovulation (SO) have a different spectral profile than those collected after a natural ovulation (NO). As with the analysis using PCA (Figure 4A), CVA shows that the SO group has greater intra-group variation than both the NO and the MIV groups (Figure 4B).

### Raman Spectroscopy Allows Direct Assessment of Oocyte Quality

Upon examination of the three methods of oocyte development, those obtained from natural ovulations (NO), after superovulation (SO), or oocytes from follicles that had been matured *in vitro* (MIV), superovulation resulted in the greatest degree of inter-oocyte spectral variance. Furthermore, the morphological appearance of oocytes from this group was also more varied than the other treatment groups, with a number of oocytes appearing to have significant morphological defects. Superovulated oocytes were, therefore, graded from 1 to 5 according to their morphological appearance (Table 1). Due to the small number of oocytes graded as Grades 3–5, and since fully mature oocytes should be Grade 1, oocytes were subsequently grouped simply as being of either good quality (Grade 1; n = 24) or poor quality (Grades 2–5; n = 35) for further analyses.

Good and poor quality SO oocytes were analysed using a two-tailed unpaired t-test, for each wavenumber examined. This analysis revealed that the intensities of 12 Raman bands were significantly different (p = 0.01) between the good and poor quality groups: in particular, greater intensities were observed in the good quality (Grade 1) oocytes at 1339, 1401, 1585, 1605, 1621 and 1658 cm^−1^, all of which correspond to protein vibrations, with the exception of the 1401 cm^−1^ peak which arises due to the presence of the symmetric carboxylate stretch in fatty acids. Conversely, good quality (Grade 1) oocytes had a reduction in intensity of the peaks occurring at 851, 888, 905, 972, 1063 and 1447 cm^−1^ when compared to those of poor quality oocytes. Some of the lower peaks are difficult to assign due to overlapping bands in this region, but possible sources could be fatty acids, saccharides or amino acid residues, whilst the 1063 cm^−1^ peak is associated with the C-C chain stretch in lipids. The change in the 1447 cm^−1^ band, which occurs as a result of C-H deformation, is attributed to lipids due to the presence of the shoulder below it and the lack of a discernible shoulder at 1460 cm^−1^ that occurs when this peak is produced by proteins.

**Figure 5:**
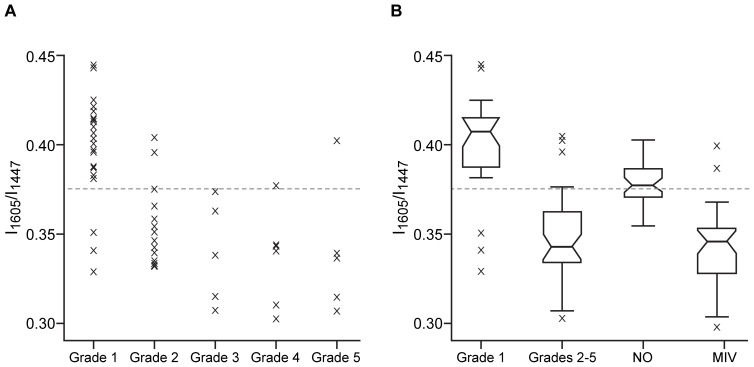
High 1605∶1447 cm ^−1^ protein-to-lipid ratio is a good indicator of oocyte quality. (**A**): Scatter plot showing the ratios of the intensity of the Raman peak located at 1605 and 1447 cm^−1^ for each mean spectrum derived from the SO (superovulated) oocyte group. Results are presented according to the grade of the oocyte, as judged by its morphological appearance. The dashed line indicates the cut-off (0.3752) as determined by analysis of the ROC curve. (**B**): Box-plot showing the ratios of the intensity at 1605 and 1447 cm^−1^ for Grade 1 (good quality; n = 24) and Grades 2–5 (poor quality; n = 35) superovulated (SO) oocytes, oocytes collected after natural ovulations (NO; n = 38) and those which had been matured *in vitro* (MIV; n = 15). Within each box-plot, the narrow line shows the median, while upper and lower lines indicate first and third quartiles, with black whiskers extending 1.5 times the inter-quartile range in both directions: data outwith of this range are considered outliers, annotated by crosses. The dashed line indicates the cut-off (0.3752) as determined by analysis of the ROC curve for the SO oocyte data presented in (A).

Given that the t-test analyses indicated overall that poor quality (Grades 2–5) oocytes displayed greater lipid content, whilst good quality (Grade 1) oocytes have a higher protein content, the ratio of protein-to-lipid in poor quality oocytes should be lower than that in good quality oocytes, potentially providing a useful assessment of oocyte quality. Consequently, all ratio combinations of the Raman bands identified by the t-tests were examined for their discriminatory power. One ratio in particular, that of 1605∶1447 cm^−1^, was found to be a powerful way in which to separate the different grades of oocytes: the peak of 1605 cm^−1^ arises due to phenylalanine, whilst that of 1447 cm^−1^ can be assigned to C-H deformation of lipids. That ratio was, therefore, utilised as a specific representation of the protein-to-lipid ratio of the oocyte, with a higher protein content indicating a better oocyte quality, and used to examine all groups of oocytes. Initially, the 1605∶1447 ratio was examined in the five grades of SO oocytes (Figure 5A). Analysis of the ROC curve for these data indicated that oocytes could be separated with the highest degree of certainty with the ratio cut-off set at 0.375, with all but three of the Grade 1 oocytes above the cut-off, equating to a sensitivity of 87.5%. Similarly, there was very little misclassification of Grade 3–5 oocytes, with all Grade 3 oocytes correctly classified and only one each of Grade 4 and Grade 5 oocytes misclassified. The specificity of the comparison between Grade 1 and Grade 2 was lower than that comparing Grade 1 with Grades 3, 4 or 5 oocytes, which is to be expected, since Grade 3–5 oocytes are likely to be more severely compromised than those belonging to Grade 2, given their poor morphological appearance.

The 1605∶1447 cm^−1^ ratio was then examined for good quality (Grade 1) SO oocytes, poor quality (Grades 2–5) SO oocytes, and for the oocytes obtained from natural ovulations (NO) and after follicle culture (MIV) (Figure 5B). The median of the NO group sits above the cut-off previously determined from the ROC analysis of Grade 1 and Grades 2–5 SO oocytes, indicating that the spectral profile of the NO group is more closely related to the Grade 1, good quality SO profile. There is also very little variability in the NO data. Strikingly, the MIV group sits below the cut-off, indicating that the profile of this group of oocytes is more similar to that of the poor quality (Grades 2–5) SO oocytes.

## Discussion

Raman spectra have been shown here to be able to discriminate between oocytes at early stages of development and developed, mature oocytes, and also to indicate oocyte quality. Furthermore, analysis of spectral profiles shows that oocytes which have developed *in vitro* are protein-deficient when compared to those matured *in vivo*. Finally, the protein-to-lipid ratio of an oocyte’s cytoplasm was found to be a good indicator of its quality.

Oocytes from follicles at an early, pre-antral stage of development (IMM; used as starting material for the follicle culture) exhibited much greater spectral variation than the oocytes that had matured *in vitro* (MIV; obtained from the end of the culture period). The source of the oocytes in each group needs to be considered when interpreting this observation. Healthy-looking, late pre-antral follicles were the originating source of oocytes in both cases. However, if left to develop *in vivo*, many follicles would have become atretic during antral stages of development. IMM oocytes contains all oocytes assigned to that group, without any further selection having taken place, and is therefore likely to include oocytes already destined for atresia. Indeed, some of the IMM group were discarded during the culture if atresia became apparent during that time. As such, the MIV follicle cohort (obtained at the end of the culture period) should represent a more uniform distribution, and the spectral variation shows this.

Immature oocytes had significantly lower spectral contributions arising from lipid vibrations when compared with oocytes matured *in vitro*. The cytoplasmic maturation that occurs in oocytes as they grow and develop involves increase in cytoplasmic organelles, and in particular the increase in mitochondria: it is possible that these double-membrane bound organelles contribute to the increase in lipid bond vibrations. Contributions are also likely to arise from the lipid droplets that accumulate throughout ooplasmic maturation [22]–[24], thought to be energy reserves for subsequent embryonic development and membrane production [7]. Depending upon the source of the marked increase in lipid contributions, the transfer of this methodology for quantification of maturity in human oocytes may not be straightforward, since the lipid droplet content of human oocytes is lower than that of mouse oocytes [25].

The development of oocytes *in vitro*, including culture of oocytes through the final maturation process, has attracted much attention, but variation in protocols has led to different opinions of its efficacy. The process of follicular growth and oocyte maturation *in vivo* is a highly orchestrated process, regulated by a number of environmental factors. Consequently, replication of this process *in vitro* has proved difficult. Significant attention has been given to the optimisation of *in vitro* growth of pre-antral follicles/ovarian tissue and subsequent maturation of oocytes in the mouse model. Whilst results suggest that development and maturation *in vitro* can support normal nuclear maturation, its ability to support cytoplasmic competency is less clear; this could explain the relatively poor fertilisation efficiencies that have been found. It has been postulated that the reduced developmental competence following *in vitro* growth may result from one or more of three possible mechanisms: the culture environment does not support cytoplasmic maturation; the protocol induces an asynchrony between nuclear and cytoplasmic maturation; or the oocytes possess an intrinsically reduced developmental potential [5]. The results of the PCA here revealed that oocytes developed *in vitro* had lower spectral contributions arising from protein vibrations than ovulated oocytes whether obtained from natural ovulations of following a superovulation regimen. These data are consistent with the findings of several authors who have reported lower protein expression in *in vitro* matured murine [26], bovine [27] and human [28] oocytes, when compared to their *in vivo* matured counterparts. In certain instances, some proteins were found to be absent in the *in vitro* developed oocytes, whilst Kim et al. [26] specifically identified significantly reduced expression of β-actin and insulin-like growth factor II.

More spectral variation exists in oocytes from the superovulated group than in those from either natural ovulations or from the group matured *in vitro*. This is of particularly strong significance given that the superovulated group contains 1.5 times the number of samples of the other groups (n = 59, a group size that should reduce the impact of outliers). The superovulated oocytes contained some oocytes with lower protein content than the naturally ovulated oocytes, but also others with a greater protein content. The superovulation protocol recruits follicles from a fairly wide range of developmental stages, which could lead to the presence of relatively immature and of post-mature oocytes in the superovulated cohort. Low protein-content oocytes may arise following asynchrony of follicle and oocyte development due to the superovulation regime, leading to the potential for resumption of meiosis prior to complete cytoplasmic maturation; superovulated oocytes with a particularly high protein content could represent post-mature oocytes, characterised by a number of factors including a large perivitelline space [29].

The large variation in the number, morphology and quality of oocytes collected after superovulation, as seen in this work, is well documented and has been reported to be strain dependent in mice [30]–[32]. Here, further work was performed to investigate whether the variation in oocyte quality could be predicted by Raman spectroscopy. Comparison of the mean spectra for good quality (Grade 1) and poor quality (Grades 2–5) oocytes displayed numerous regions of significant difference: specifically, Grade 1 oocytes displayed increased intensities associated with protein vibrations and lower intensities resulting from lipids and saccharides, when compared to oocytes judged as being of poor quality.

Examination of various ratios of the significantly different peaks yielded a ratio which provided a good separation of the oocyte qualities: the ratio of the peak arising due to phenylalanine at 1605 cm^−1^ and the peak at 1447 cm^−1^ assigned to C-H deformation of lipids. This ratio represented the proportion of protein-to-lipids in the oocyte: the higher the ratio, the better the oocyte quality. These findings concur with previous results indicating that good quality oocytes have high protein content. Results also indicate that poor quality oocytes have a high lipid content, which would result in a lower protein-to-lipid ratio. As most of the poor quality (Grades 2–5) oocytes had cytoplasmic granularity to some extent (see Table 1), this could be the source of the increased lipid content, as has been found previously [18]. Given this, the work here provides proof of principle that oocyte quality can be discerned through analysis of protein-to-lipid ratio in the oocyte, with the 1605∶1447 cm^−1^ ratio particularly effective for such an analysis. Raman spectroscopy could provide a specific analysis of the 1605∶1447 cm^−1^ ratio rapidly, potentially allowing it to be undertaken on unfixed oocytes, prior to fertilisation *in vitro*.

To translate this proof of principle to clinical utility will require further work. For the use of the oocytes after quality assessment, the method has to be substantiated for application to live cells. To achieve this will require a significant reduction in the time required for spectral acquisition on the sample [33]. For the present work we sequentially reduced our spectral sampling to 101 positions across the cell’s midline from the 1610 we have used in previous imaging studies [19]; we regard 101 positions to be a minimum sample set to give a robust averaging of the cell’s biomolecular composition. Alternately, we can speed Raman data acquisition at each point by sampling only the Raman peaks of interest (namely 1605 and 1447 cm^−1^): indeed, this could be further accelerated through the use of resonant Raman excitation employed in techniques such as coherent anti-Stokes Raman scattering microscopy (CARS) [34].

In conclusion, the work presented here demonstrates that immature and mature oocytes have distinct cytoplasmic spectral profiles. Furthermore, results show that a logistic regression model constructed using Raman intensity ratios can be used to successfully classify oocyte maturity. Raman micro-spectroscopy used in combination with PCA and CVA also revealed differences between oocytes from fully grown follicles, depending on the environment in which follicle development had occurred: unstimulated and superovulated oocytes possess ooplasm with similar biochemical composition, whilst oocytes that have developed *in vitro* have lower protein content, consistent with incomplete cytoplasmic maturation. Finally, the quality of superovulated oocytes can be distinguished by the ratio of protein to lipid in their ooplasm. These results indicate that Raman spectroscopy is a powerful technique for the analysis of oocyte maturation, ovulation mechanism and quality. In particular, the implementation of a Raman imaging protocol used in combination with the automated pre-processing and analysis algorithms which have been developed would benefit future work by enabling rapid live-cell analysis, allowing testing of unfixed oocytes so that only high quality oocytes are subsequently fertilized.

## Supporting Information

Figure S1 **Raman Microspectrometer.**
(PDF)

Figure S2 **Peak Assignments Summary Illustration.**
(PDF)

Figure S3 **Fixation Method Study.**
(PDF)

Text S1 **Statistical Analyses.**
(PDF)

## References

[1] Picton H, Briggs D, Gosden R (1998) The Molecular Basis of Oocyte Growth and Development. Mol Cell Endcrinol 145: 27–37. 10.1016/s0303-7207(98)00166-x 9922096

[2] Telfer EE (2007) McLaughlin (2007) M. Rep Biomed Online. 15: 288–295. 10.1016/s1472-6483(10)60341-0 17854526

[3] Gosden R (1995) Oocyte Development Through Life. In: Grudzinskas JG, Yovich JL, editors. Gametes - The Oocyte. Cambridge: Cambridge University Press. 119–149.

[4] Baker SJ, Spears N (1999) The Role of Intra-Ovarian Interactions in the Regulation of Follicle Dominance. Hum Reprod Update 5: 153–165. 10336019 10.1093/humupd/5.2.153

[5] Combelles CM, Cekleniak NA, Racowsky C, Albertini DF (2002) Assessment of nuclear and cytoplasmic maturation in in-vitro matured human oocytes. Hum Reprod 17: 1006–1016. 11925398 10.1093/humrep/17.4.1006

[6] Murray AA, Swales AK, Smith RE, Molinek MD, Hillier S, et al (2008) Follicular growth and oocyte competence in the in vitro cultured mouse follicle: effects of gonadotrophins and steroids. Mol Hum Reprod 14: 75–83. 18204068 10.1093/molehr/gam092

[7] Gosden R, Bownes M (1995) Molecular and cellular aspects of oocyte development. In: Grudzinskas JG, Yovich JL, editors. Gametes - The Oocyte. Cambridge: Cambridge University Press. 23–35.

[8] Braga DP, Savio Figueria RD, Rodrigues D, Madaschi C, Pasqualotto FF, et al (2007) Prognostic value of meiotic spindle imaging on fertilization rate and embryo development in in vitro matured human oocytes. Fertil Steril 90: 429–433. 17953959 10.1016/j.fertnstert.2007.06.088

[9] Madaschi C, de Souza Bonetti TC, de Almeida Ferreira Braga DP, Pasqualotto FF, Iaconelli A Jr, et al (2008) Spindle imaging: a marker for embryo development and implantation. Fertil Steril 90: 194–198. 17727849 10.1016/j.fertnstert.2007.05.071

[10] WangWH, Meng L, Hackett RLK, Keefe DL (2001) Developmental ability of human oocytes with or without birefringent spindles imaged by Polscope before insemination. Hum Reprod 16: 1464–1468. 11425830 10.1093/humrep/16.7.1464

[11] Moon JH, Hyun CS, Lee SW, Son WY, Yoon SH, et al (2003) Visualization of the metaphase II meiotic spindle in living human oocytes using the Polscope enables the prediction of embryonic developmental competence after ICSI. Hum Reprod 18: 817–820. 12660277 10.1093/humrep/deg165

[12] Amharref N, Beljebbar A, Dukic S, Venteo L, Schneider L, et al (2007) Discriminating healthy from tumor and necrosis tissue in rat brain tissue samples by Raman spectral imaging. Biochim Biophys Acta 1768: 2605–2615. 17761139 10.1016/j.bbamem.2007.06.032

[13] Chan JW, Taylor DS, Zwerdling T, Lane SM, Ihara K, et al (2006) Micro-Raman spectroscopy detects individual neoplastic and normal hematopoietic cells. Biophysical Journal 90: 648–656. 16239327 10.1529/biophysj.105.066761 PMC1367069

[14] Haka AS, Shafer-Peltier KE, Fitzmaurice M, Crowe J, Dasari RR, et al (2005) Diagnosing Breast Cancer by Using Raman Spectroscopy. Proc Natl Acad Sc 102: 12371–12376. 16116095 10.1073/pnas.0501390102 PMC1194905

[15] Jess PR, Smith DD, Mazilu M, Dholakia K, Riches AC, et al (2007) Early detection of cervical neoplasia by Raman spectroscopy. Int J Cancer 121: 2723–2728. 17724716 10.1002/ijc.23046

[16] Scott R, Seli E, Miller K, Sakkas D, Scott K, et al (2008) Noninvasive metabolomic profiling of human embryo culture media using Raman spectroscopy predicts embryonic reproductive potential: a prospective blinded pilot study. Fertil Steril 90: 77–83. 18281045 10.1016/j.fertnstert.2007.11.058

[17] Seli E, Sakkas D, Scott R, Kwok SC, Rosendahl SM, et al (2007) Noninvasive Metabolomic Profiling of Human Embryo Culture Media Using Raman Spectroscopy Predicts Embryonic Reproductive Potential: Prospective Blinded Pilot Study. Fertil Steril 88: 1350–1357. 18281045 10.1016/j.fertnstert.2007.11.058

[18] Wood BR, Chernenko T, Matthäus C, Diem M, Chong C, et al (2008) Shedding new light on the molecular architecture of oocytes using a combination of synchrotron Fourier transform-infrared and Raman spectroscopic mapping. Anal Chem 80: 9065–9072. 18983174 10.1021/ac8015483 PMC2761072

[19] Davidson B, Spears N, Murray A, Elfick APD (2012) The Changing Biochemical Composition and Organisation of the Murine Oocyte and Early Embryo as Revealed by Raman Spectroscopic Imaging. J Raman Spectrosc 43: 24–31.

[20] Spears N, Boland NI, Murray AA, Gosden RG (1994) Mouse oocytes derived from *in vitro* grown primary ovarian follicles are fertile. Human Reprod 9: 527–532. 10.1093/oxfordjournals.humrep.a138539 8006146

[21] Maquelin K, Choo-Smith LP, van Vreeswijk T, Endtz HP, Smith B, et al (2000) Raman spectroscopic method for identification of clinically relevant microorganisms growing on solid culture medium. Anal Chem 72: 12–19. 10655628 10.1021/ac991011h

[22] Sathananthan AH (1994) Ultrastructural changes during meiotic maturation in mammalian oocytes: unique aspects of the human oocyte. Microsc Res Tech 27: 145–164. 8123907 10.1002/jemt.1070270208

[23] Niimura S, Kawakami SY, Takano H (2004) Changes in the Amount of Cytoplasmic Inclusions in Mouse Oocytes During Meiotic Maturation In Vivo and In Vitro. Reprod Med Biol 3: 231–236. 10.1111/j.1447-0578.2004.00075.x PMC5906822 29699201

[24] Ami D, Mereghetti P, Natalello A, Doglia SM, Zanoni M, et al (2011) FTIR spectral signatures of mouse antral oocytes: molecular markers of oocyte maturation and developmental competence. Biochem Biophys Acta 1813: 1220–1229. 21435359 10.1016/j.bbamcr.2011.03.009

[25] Borini A, Coticchio G (2009) The human oocyte: controlled rate cooling. In: Gardner DK, Weissman A, Howles CM, Shoham Z, editors. Textbook of assisted reproductive technologies: laboratory and clinical perspectives. London: Informa Healthcare. pp. 255–274.

[26] Kim DH, Ko DS, Lee HC, Lee HJ, Park WI, et al (2004) Comparison of maturation, fertilization, development, and gene expression of mouse oocytes grown in vitro and in vivo. J Assist Reprod Genet 21: 233–240. 15526980 10.1023/B:JARG.0000042008.83699.cc PMC3455184

[27] Kastrop PM, Bevers MM, Destree OH, Kruip TA (1991) Protein synthesis and phosphorylation patterns of bovine oocytes maturing in vivo. Mol Reprod Dev 29: 271–275. 1931043 10.1002/mrd.1080290309

[28] Trounson A, Anderiesz C, Jones G (2001) Maturation of human oocytes in vitro and their developmental competence. Reprod 121: 51–75. 10.1530/rep.0.1210051 11226029

[29] Miao YL, Kikuchi K, Sun QY, Schatten H (2009) Oocyte aging: cellular and molecular changes, developmental potential and reversal possibility. Hum Reprod Update 15: 573–585. 19429634 10.1093/humupd/dmp014

[30] Byers SL, Payson SJ, Taft RA (2006) Performance of ten inbred mouse strains following assisted reproductive technologies. Theriogenol 65: 1716–1726. 10.1016/j.theriogenology.2005.09.016 16271754

[31] Nagy A, Gertsenstein M, Vintersten K, Behringer R (2003) Manipulating the Mouse Embryo: A Laboratory Manual. Cold Spring Harbor: Cold Spring Harbor Press. 764p.

[32] Vergara GJ, Irwin MH, Moffat RJ, Pinkert CA (1997) In vitro fertilization in mice: strain differences in response to superovulation protocols and effect of cumulus cell removal. Theriogenol 47: 1245–1252. 10.1016/s0093-691x(97)00104-0 16728073

[33] Schultz RM (2007) Of light and mouse embryos: less is more. PNAS 104: 14547–14548. 17785409 10.1073/pnas.0707142104 PMC1976224

[34] Downes A, Mouras R, Bagnaninchi P, Elfick A (2011) Raman spectroscopy and CARS microscopy of stem cells and their derivatives. J Raman Spectrosc 42: 1864–1870. 22319014 10.1002/jrs.2975 PMC3272468

