# Supplementary material for: Raman Micro-Spectroscopy Can Be Used to Investigate the Developmental Stage of the Mouse Oocyte
Source: PLoS One. 2013 Jul 1;8(7):e67972. doi: 10.1371/journal.pone.0067972 (PMC3698144; doi:10.1371/journal.pone.0067972)
Supplement: Figure S1 — Raman Microspectrometer. (PDF) [file pone.0067972.s001.pdf]

__**Supporting Information.**__

Raman micro-spectroscopy can be used to investigate the developmental stage of the mouse oocyte.

Davidson, Murray, Elfick and Spears

__**Figure S1: Raman Microspectrometer**__

A schematic diagram of the Renishaw InVia Raman microscope depicting the key optical components and paths with the major components is shown in Figure S1. The spectral window 450 - 1790 cm^{-1} captured using SynchroScan mode in which the diffraction grating is scanned so as to facilitate high spectral resolution; the spectral resolution achieved by the 1200 lines/mm grating was ~1 cm^{-1}.

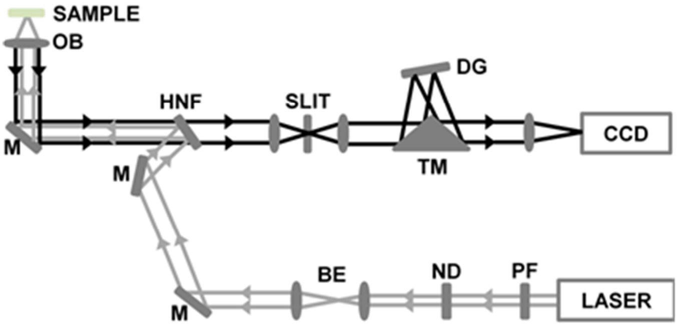

Figure S1: *Schematic of the Raman microspectrometer: BE, beam expander; DG, diffraction grating; HNF, holographic notch filter; M, mirror; ND, neutral density filter; OB, microscope objective; PF, plasma filter; and TM, triangular mirror. The incident laser and scattered light paths are depicted by the grey and black lines, respectively.*
