# Supplementary material for: Raman Micro-Spectroscopy Can Be Used to Investigate the Developmental Stage of the Mouse Oocyte
Source: PLoS One. 2013 Jul 1;8(7):e67972. doi: 10.1371/journal.pone.0067972 (PMC3698144; doi:10.1371/journal.pone.0067972)
Supplement: Figure S2 — Peak Assignments Summary Illustration. (PDF) [file pone.0067972.s002.pdf]

<u>Supporting Information.</u>

Raman micro-spectroscopy can be used to investigate the developmental stage of the mouse oocyte.

Davidson, Murray, Elfick and Spears

<u>Figure S2: Peak Assignments Summary Illustration</u>

Within a Raman spectrum there may exist a number of peaks each associated with differing modes of vibration for a particular chemical moiety. Assignment of bond vibrations is well established in the literature. A summary diagram depicting the position of many biologically relevant bond vibrations is shown in Figure S2. De Gelder *et al*. (2007) have published thorough study creating a library of spectra for biological molecules.

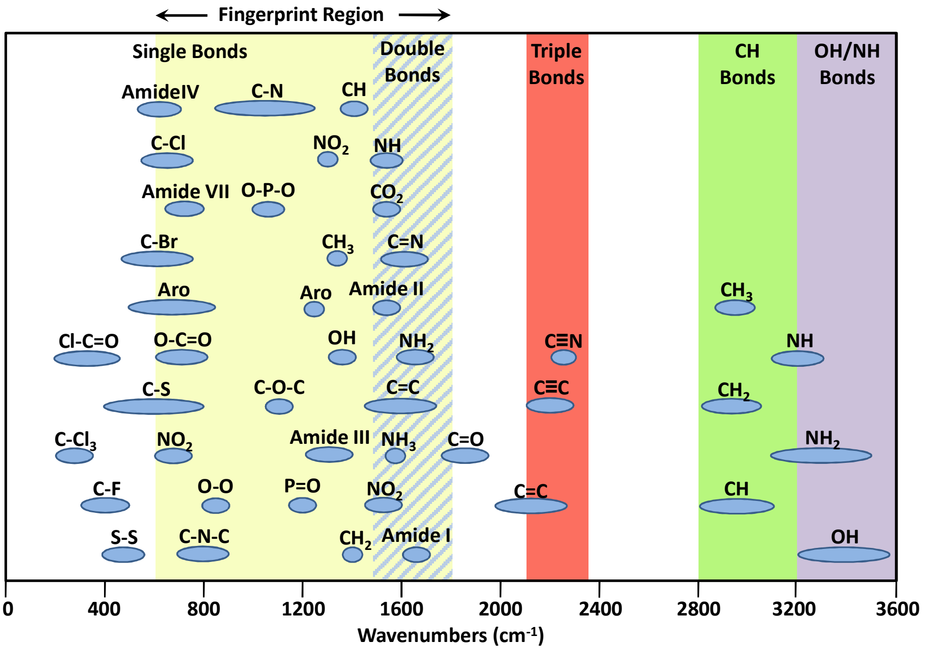

Figure S2: *Schematic diagram showing the position of peak assignments (blue ovals) for various chemical moieties of biological relevance.*

Reference:

J. De Gelder, K. De Gussem, P. Vandenabeele, L. Moens, Reference database of Raman spectra of biological molecules, Journal of Raman Spectroscopy 38 (2007) 1133–1147.
