# Supplementary material for: Raman Micro-Spectroscopy Can Be Used to Investigate the Developmental Stage of the Mouse Oocyte
Source: PLoS One. 2013 Jul 1;8(7):e67972. doi: 10.1371/journal.pone.0067972 (PMC3698144; doi:10.1371/journal.pone.0067972)
Supplement: Figure S3 — Fixation Method Study. (PDF) [file pone.0067972.s003.pdf]

Supporting Information.

Raman micro-spectroscopy can be used to investigate the developmental stage of the mouse oocyte.

Davidson, Murray, Elfick and Spears

Figure S3: Fixation Method Study

The effect of fixation on the oocyte spectrum was investigated for different fixatives protocols: 2.5% glutaraldehyde and 4% paraformaldehyde fixation was carried out for 30 minutes at room temperature, whilst the ethanol fixation was performed by passing the oocytes through 20, 30, 40, 50 and 70 % ethanol solutions for 5 minutes each. After fixation, oocytes were rinsed and resuspended in PBS. Unfixed oocytes were washed in PBS and used immediately. Ten oocytes were processed by each method with data analysis conducted using PCA, see below. The resultant biplot clearly shows paraformaldehyde fixation to most closely model the unfixed oocytes.

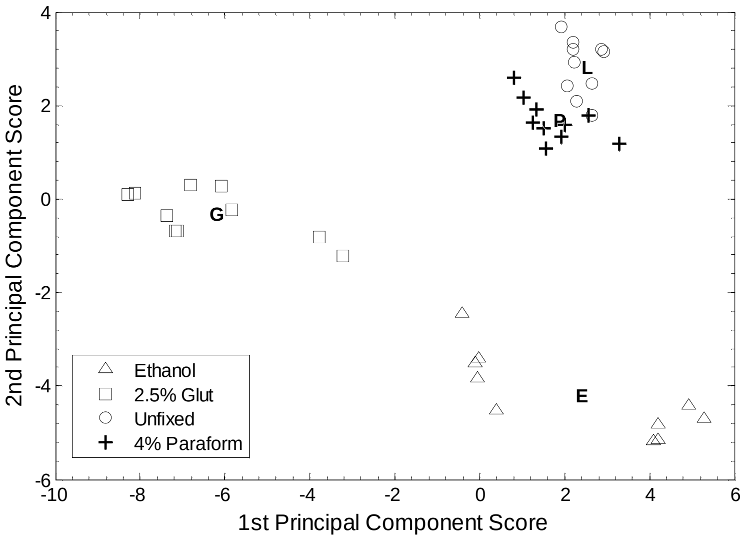

Figure S3: *Biplot showing the scores of each datum against the first and second component axes generated during the PCA. The mean score for the ethanol, glutaraldehyde and paraformaldehyde fixed groups and the unfixed group are denoted by E, G, P and L, respectively.*
