# Supplementary material for: Raman Micro-Spectroscopy Can Be Used to Investigate the Developmental Stage of the Mouse Oocyte
Source: PLoS One. 2013 Jul 1;8(7):e67972. doi: 10.1371/journal.pone.0067972 (PMC3698144; doi:10.1371/journal.pone.0067972)
Supplement: Text S1 — Statistical Analyses. (PDF) [file pone.0067972.s004.pdf]

Supporting Information.

Raman micro-spectroscopy can be used to investigate the developmental stage of the mouse oocyte.

Davidson, Murray, Elfick and Spears

Text S1: Statistical Analyses

Logistic Regression

A logistic regression was also performed using the standardised intensities of three Raman peaks that had been found to be significantly different by t-test (p=0.001). The intensity of these peaks were standardised by calculating the ratio with respect to the phenylalanine stretch peak at 1003 cm^{-1} wavenumbers, as this peak showed no significant variation between groups. Unlike linear regression which fits the measured data using a linear relationship, logistic regression uses the non-linear logit link function, as described by Equation 1, which ensures that the outcome is constrained to values between 0 and 1.

$$\ln\left(\frac{p}{p-1}\right)=\beta_0+\beta_1X_1+\beta_2X_2+...+\beta_nX_n$$

In Equation 1, p represents the probability that a particular oocyte belongs to the mature group (i.e. y = 1). As such, the left hand side of the equation is equivalent to the log odds of the oocyte belonging to the mature group. $\beta_0, \beta_1, \beta_2$ and β_n represent the derived coefficients determined for the independent variables, which in this case are the intensity ratios as defined by X_1, X_2 and X_n.

Principal Component Analysis (PCA)

Principal component analysis uses an eigen decomposition of the covariance matrix to construct a rotated set of orthogonal axes in the variable space such that the first axis
or principal component – is oriented in the direction of greatest variability in the data, the second principal component (PC) shows the highest variability for all directions orthogonal to the first PC, and so forth. Cross-plotting the data along the PC axes tends to reveal more about the variability of the data than plots in the original variable space.

Canonical Variates Analysis (CVA)

CVA seeks to identify linear combinations that uncover differences between groups. This is achieved by performing a generalized eigen decomposition to identify eigenvalues and vectors of the between-groups covariance matrix, *B*. The within-groups covariance matrix is the pooled covariance matrix, *S*. The between-groups covariance matrix describes the variation of the group means about the global mean. It may be given by

$$B=\frac{K}{n(K-1)}\sum_{k=1}^{K}n_k\left(\bar{X}_k-\bar{X}\right)\left(\bar{X}_k-\bar{X}\right)'$$

where *K* is the number of groups, *n* is the total number of data, n_k is the number of data in group *k*, $\bar{X}_k$ is the mean vector for group *k* and $\bar{X}$ is the global mean vector.

If u_1 is the first eigenvector, that associated with the largest eigenvalue of $S^{-1}B$, then the first canonical variate, $v_1 = u_1'x$, is the linear combination of variables that shows the maximum ratio of between-groups to within-groups variation. Interpretation of results proceeds as for PCA, with "ratio of between- to within-groups" variation substituted for "total variation".
